# Myocardial oxygenation in hibernating myocardium: insights from blood oxygen level dependent imaging pre and post revascularization

**DOI:** 10.1186/1532-429X-16-S1-P176

**Published:** 2014-01-16

**Authors:** Suchi Grover, Darryl Leong, Craig Bradbrook, Angela Walls, Jawad Mazhar, Joseph Selvanayagam

**Affiliations:** 1 Flinders University, Adelaide, South Australia, Australia; 2 Cardiology, Flinders Medical Centre, Adelaide, South Australia, Australia; 3 Flinders Cardiac Cardiovascular Magnetic Resonance Imaging, Flinders Medical Centre, Adelaide, South Australia, Australia

## Background

The mechanisms underlying hibernating myocardium are controversial. Whether de-oxygenation occurs at rest and/or stress in severely dysfunctional viable (i.e. hibernating) myocardium is currently unknown. By utlising the paramagnetic properties of deoxy-haemoglobin, blood oxygen level dependent magnetic resonance imaging (BOLD-MRI) can detect oxygenation and myocardial ischemia. We applied BOLD imaging to examine the pathophysiological relationship between coronary stenosis, regional wall motion abnormalities, ventricular scar and myocardial oxygenation in hibernating myocardium.

## Methods

BOLD imaging was acquired at rest and stress after 5 minutes of administration of adenosine (140 μg/kg/min) and assessed quantitatively (using a BOLD signal intensity index [stress/resting signal intensity] in 11 patients pre and median of 8 months (207, 299 days) post revascularisation. Late gadolinium (LGE) images were acquired using a T1 weighted segmented inversion recovery sequence and regional wall motion abnormalities (RWM) were scored pre and post revascularization using standard criteria. All segments were matched for BOLD, RWM and LGE analysis. Significant stenosis were classed as >50% by QCA.

## Results

BOLD signal intensity (SI) for the overall group improved from -5.1 ± 17.0 at baseline to 4.5 ± 11.2%, post revascularization (p = 0.001). 32% of segments evaluated did not have a significant coronary stenosis, 45% had significant stenosis and 23% had stenosis with collaterals. There was an inverse relationship between delta BOLD SI (difference between stress and rest BOLD SI) and the presence of significant coronary stenosis (p = 0.03). Post revascularization, there was an improvement in the RWM score (p < 0.001). 72% of total myocardial segments were dysfunctional (RWM score ≥1) before revascularization, and 46% of segments improved contraction post revascularisation. Significantly, segments that showed an improvement in RWM score of ≥1 post-revascularization ('hibernating segments') also demonstrated a significant improvement in delta BOLD SI from -3.7 ± 11.2 to 4.5 ± 14, p = 0.03. There was no change when we excluded segments with >75% transmural hyperenhancement.

## Conclusions

The noninvasive assessment of myocardial oxygenation by BOLD imaging offers valuable insights into the pathophysiology of hibernating myocardium. Although preliminary, our results indicate that the BOLD response is impaired in hibernating myocardium and that oxygenation is not down regulated in these myocardial segments.

## Funding

This project was funded by National Health and Research Medical Council, Australia.

